# Vitamin E intake and multiple health outcomes: an umbrella review

**DOI:** 10.3389/fpubh.2023.1035674

**Published:** 2023-07-13

**Authors:** Tianyi Zhang, Xianyanling Yi, Jin Li, Xiaonan Zheng, Hang Xu, Dazhou Liao, Jianzhong Ai

**Affiliations:** Department of Urology, West China Hospital, Institute of Urology, Sichuan University, Chengdu, China

**Keywords:** vitamin E, health outcomes, umbrella review, meta-analyses, intake

## Abstract

**Background:**

The benefits of vitamin E (VE) for multiple health outcomes have been well evaluated in many recent studies.

**Objective:**

The purpose of this umbrella review was to conduct a systematic evaluation of the possible associations between VE intake and various health outcomes.

**Methods:**

We systematically searched various databases, such as PubMed, Embase, and the Web of Science, to identify related meta-analyses of observational studies and randomized trials. We estimated the effect size of each association by using the random or fixed effects models and the 95% confidence intervals. We used standard approaches to evaluate the quality of the articles (AMSTAR) and classified the evidence into different levels of quality (GRADE).

**Results:**

A total of 1,974 review articles were searched, and 27 articles with 28 health outcomes were yielded according to our exclusion criteria. The intake of VE was inversely associated with the risk of breast cancer, lung cancer, esophageal cancer, gastric cancer, pancreatic cancer, kidney cancer, bladder cancer, cervical neoplasms, cardiovascular disease, Parkinson's disease, depression, age-related cataracts, metabolic syndrome, and fracture. Overall, most of the quality of the evidence was low or very low. Three outcomes (stroke, age-related cataracts, obesity) were identified as having a “moderate” level of quality. The AMSTAR scores for all health outcomes ranged from 5 to 10.

**Conclusion:**

Our study revealed that VE intake is beneficially related to multiple health outcomes. However, future studies on recommended doses and recommended populations of VE are also needed.

**Systematic review registration:**

http://www.crd.york.ac.uk/PROSPERO/, identifier: CRD42022339571.

## 1. Introduction

Vitamin E (VE), a fat-soluble antioxidant, is composed of tocopherol and tocotrienol α, β, δ, γ subtypes (1). Previous studies have found a potential link between VE and many diseases (2). Since the burden of morbidity and mortality from chronic diseases and cancer is increasing, VE has been widely studied as a potential preventive measure. Oxidative stress is considered a central mechanism of carcinogenesis and is an important process in many diseases, and VE generally helps prevent multiple diseases that are caused by oxidative damage (3, 4). The effects of VE on different types of cancers (such as breast, stomach, and bladder cancer), cardiovascular diseases, and neurological disorders may all be related to it. The possible mechanisms of carcinogenesis are as follows: (1) prevention of DNA damage through scavenging lipid hydrogen peroxide radicals; (2) protection of the nerves from free radical-mediated damage; (3) repression of the protein kinase C (PKC) pathway and enhancement of immune system function; (4) inhibition of cell cycle progression and cell proliferation via reduction of cyclin D1 and cyclin E; and (5) decrease in the expression of cyclooxygenase-2 and 8-hydroxydeoxyguanosine and type I insulin-like growth factor receptor to inhibit peroxidation and induce cell apoptosis, leading to suppression of cell proliferation (5–7). The association between VE and various health outcomes has been evaluated in a large cohort study, a case-control study, and randomized controlled trials. The results of these studies are summarized by systematic reviews and meta-analyses. However, a comprehensive review of the association between VE and multiple health outcomes (cancer and non-cancer outcomes) has been published. An umbrella review is a popular method for systematically assessing evidence from multiple sources and may be useful in assessing potential biases in the relationship between exposure and outcomes (2, 8, 9). Therefore, we conducted this study to provide a comprehensive review for investigating the relationship between VE and health outcomes reported in published systematic reviews and meta-analyses and to further assess the validity and quality of the available evidence.

## 2. Methods

### 2.1. Umbrella review methods

We systematically searched, organized, and evaluated existing evidence from numerous systematic reviews and meta-analyses on multiple health outcomes associated with VE intake (10). We included only those systematic reviews in our study that had incorporated meta-analyses. This umbrella review was registered in PROSPERO (CRD42022339571).

### 2.2. Literature search and eligibility criteria

We searched systematic reviews and meta-analyses of observational studies and randomized trials from PubMed, Embase, and Web of Science databases from inception to March 2022. The search strategy we used was as follows: ((((((vitamin E[Title/Abstract]) OR (Tocopherol[Title/Abstract])) OR (alpha-Tocopherol [Title/Abstract])) OR (beta-Tocopherol[Title/Abstract])) OR (gamma-Tocopherol[Title/Abstract])) OR (Tocotrienol[Title/Abstract])) AND (((systematic review[Title/Abstract]) OR (meta-analysis[Title/Abstract])) OR (systematic overview[Title/Abstract])). Meta-analyses and systematic reviews with meta-analyses of observational (cohort and case-control) and interventional (randomized and nonrandomized controlled trials) studies that evaluated VE intake and health outcomes in humans were included regardless of the race, gender, country, or region of participants. If two or more health outcomes existed in a single article, data for each outcome were extracted separately. If two or more meta-analyses revealed the same association, we chose the largest one to avoid duplicate assessments. Furthermore, articles reporting VE intake with therapeutic utilities were excluded only if nontherapeutic intake was also reported. Articles written in languages other than English and not involving humans were also excluded.

### 2.3. Data extraction

The following information was extracted independently by two investigators: (1) name of the first author, (2) cancer outcomes and non-cancer outcomes, (3) year of publication, (4) category of exposure (dietary and supplement VE), (5) the number of included studies, (6) the number of events and total participants in each study, (7) study design [case-control, cohort, randomized controlled trial (RCT)], (8) type of comparisons (highest v lowest dose reduction of any dietary and supplement VE), (9) the estimated summary effect (RR, relative risk; OR, odds ratio; SMD, standard mean difference) and corresponding 95% confidence intervals (CIs), (10) type of effect model (fixed or random model), and (11) *P*-value and publication bias by Egger's test. Any difference was resolved by the third investigator.

### 2.4. Quality of included studies and quality of evidence

The AMSTAR items (a reliable strategy for evaluating the quality of system reviews and meta-analyses) were used to evaluate the quality of the included articles (11). In our umbrella review, the Grading of Recommendations, Assessment, Development, and Evaluation (GRADE) approach was used to assess the strength of the evidence and categorize it to grade different levels of quality (“high,” “moderate,” “low,” and “very low”) (12).

### 2.5. Data analysis

A summary effect size was presented with a 95% confidence interval through the fixed or random effects models reported in the meta-analysis, if available. If both the cohort and case-control studies existed in the same article, the data were extracted separately. Publication bias was assessed. I^2^ statistics and Cochran's Q test were used to estimate the heterogeneity between studies (13, 14). The results of Egger's and heterogeneity tests were significantly higher than those of the control group when the *p* < 0.10. For other tests, a *p* < 0.05 was considered significant.

## 3. Results

### 3.1. Characteristics of the included meta-analysis

**Figure 1:**
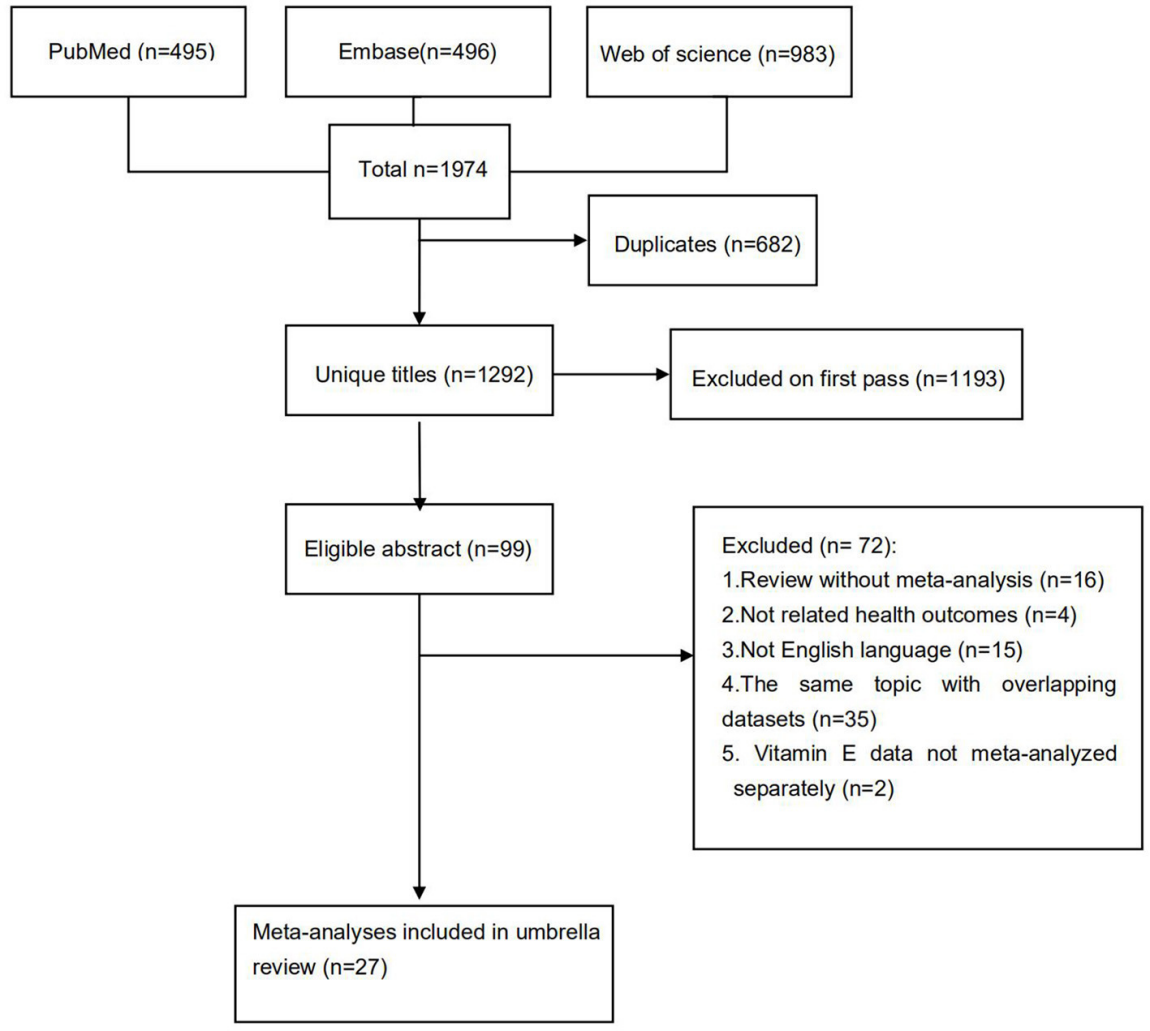
Flowchart of the systematic search and selection process.

**Figure 2:**
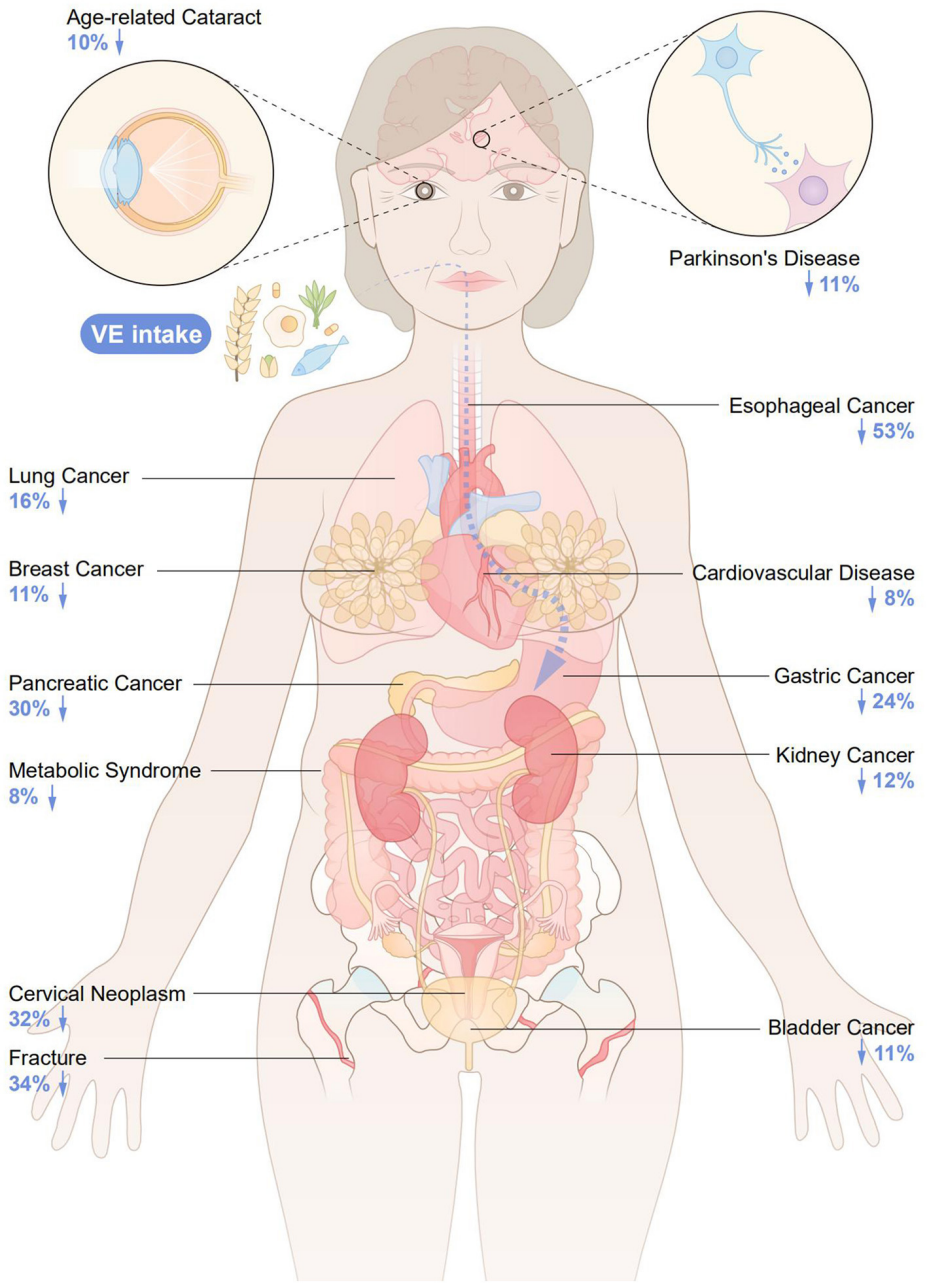
The associations between VE and multiple health outcomes.

The flowchart of the detailed selection process is presented in Figure 1. Overall, after a systematic search, a total of 1,974 articles were identified, and 27 meta-analyses with 28 health outcomes (including 13 cancer-related and 16 non-cancer-related outcomes) were enrolled according to our exclusion criteria. The associations between VE and multiple health outcomes are shown in Figure 2, and more details are presented in Table 1 (non-cancer outcomes) and Table 2 (cancer outcomes). The assessments of AMSTAR scores and GRADE classification are shown in Table 3.

**Table 1:** Associations between Vitamin E intake and non-cancer outcomes.

**Outcome**	**Category**	**References**	**No. of cases/ total**	**No. of studies**	**Cohort**	**Case-control**	**RCT**	**Meta metric**	**Effects model**	**Estimates**	**95%CI**	**I^2^; Q test *P*-value**	**Egger's test *P*- value**
**Significant associations**													
Cardiovascular Disease	Vitamin E supplements	Han et al. (15)	7,852/ 233,310	10	0	0	10	RR	Random	0.92	0.46,0.95	55.5%; 0.244	NA
Parkinson's Disease	Dietary vitamin E	Talebi et al. (16)	3,444/ 316,405	7	7	0	0	RR	Random	0.84	0.71,0.99	51.9%; 0.244	NA
Depression	Dietary vitamin E	Lee et al. (17)	187/ 354	7	0	0	7	SMD	Random	−0.88	−1.54,−0.21	87.0%; < 0.001	NA
Age-related Cataract	Dietary vitamin E	Jiang et al. (18)	NA/ 42,147	6	6	0	0	RR	Fixed	0.90	0.80,1.00	25.4%; 0.244	0.016
Metabolic Syndrome	Dietary vitamin E	Zhang et al. (19)	NA/ 51,276	10	NA	NA	NA	RR	Random	0.92	0.85,1.00	67.1%; < 0.001	NA
Fracture	Dietary vitamin E	Zhou et al. (20)	14,738/ 62,571	1	1	0	0	RR	Random	0.66	0.46,0.95	94.2%; 0.00	0.447
**Non-significant associations**													
Stroke	Vitamin E supplements	Loh et al. (21)	74,000/148,016	18	0	0	18	RR	Random	0.98	0.92,1.04	0.0%; 0.390	0.251
Parkinson's Disease	Dietary vitamin E	Talebi et al. (16)	1,024 / 2,604	5	0	5	0	RR	Random	0.80	0.57,1.12	23.4%; 0.262	NA
Alzheimer's Disease	Vitamin E supplements	Wang et al. (22)	1,313/ 13,311	5	5	0	0	RR	Random	0.81	0.53,1.33	69.2%; 0.012	0.659
Anxiety	Dietary vitamin E	Lee et al. (17)	153/ 306	5	0	0	5	SMD	Random	−0.86	−2.11,0.40	94.1%; < 0.001	NA
Glaucoma	Dietary vitamin E	Han and Fu (23)	1,262/ 244,254	5	5	0	0	OR	Fixed	0.91	0.71,1.16	25.0%; 0.250	NA
Age-related cataract	Dietary vitamin E	Jiang et al. (18)	NA/ 92,243	6	0	0	6	RR	Fixed	0.97	0.91,1.03	0.0%; 0.937	0.016
Obesity	Vitamin E supplements	Emami (24)	NA/ 1,129	21	0	0	21	WMD	Random	0.04	−0.29,0.37	0.0%; 0.999	0.384
All-Cause Mortality	Dietary vitamin E	Jayedi et al. (25)	22,823/386,854	11	9	0	2	RR	Random	0.95	0.90,1.01	48.8%; 0.030	0.460

CI, confidence interval; RCT, randomized controlled trial; RR, relative risk; OR, odds ratio; SMD, standardized mean difference; MD, mean differences; WMD, weighted mean difference; NA, not available.

**Table 2:** Associations between Vitamin E intake and cancer outcomes.

**Outcome**	**Category**	**References**	**No. of cases/ total**	**No. of studies**	**Cohort**	**Case-control**	**RCT**	**Meta metric**	**Effects model**	**Estimates**	**95%CI**	**I^2^; Q test *P* value**	**Egger test P value**
**Significant associations**													
Breast Cancer	total intake vitamin E	Fulan et al. (26)	NA/ NA	43	14	26	3	OR	Random	0.89	0.81,0.97	68.3%; NA	0.180
Breast Cancer	dietary vitamin E	Fulan et al. (26)	NA/ NA	29	9	20	0	OR	Random	0.82	0.73,0.91	72.1%; NA	0.150
Lung Cancer	dietary vitamin E	Zhu et al. (27)	4,164/ 435,532	9	9	0	0	RR	Fixed	0.84	0.76,0.93	41.1%; 0.075	0.246
Esophageal Cancer	dietary vitamin E	Cui et al. (28)	3,013/ 11,384	12	1	11	0	RR	Random	0.47	0.36,0.60	67.5%; < 0.001	0.008
Gastric Cancer	dietary vitamin E	Kong et al. (29)	3,299/ 634,667	8	3	5	0	RR	Random	0.76	0.67,0.85	43.0%; 0.090	0.254
Pancreatic Cancer	dietary vitamin E	Chen et al. (30)	3,070/ 230,206	11	4	7	0	OR	Fixed	0.70	0.62,0.81	0.0%; 0.455	0.596
Kidney Cancer	dietary vitamin E	Shen et al. (31)	1,213/ 450,463	6	6	0	0	RR	NA	0.88	0.72,1.08	49.2%; 0.023	0.928
Bladder Cancer	dietary vitamin E	Lin et al. (32)	3,265/ 575,601	11	8	0	3	RR	Fixed	0.89	0.78,1.00	19.9%; 0.254	0.707
Cervical Neoplasm	total intake vitamin E	Hu et al. (33)	NA/ 5,301	6	0	6	0	OR	Random	0.68	0.49,0.94	70.0%; 0.005	0.530
**Non-significant associations**													
Glioma	dietary vitamin E	Qin et al. (34)	3180/ NA	12	2	10	0	RR	Random	0.88	0.69,1.12	64.9%; 0.001	NA
Thyroid Cancer	dietary vitamin E	Zhang et al. (35)	1,021/ 15,005	4	0	3	1	OR	NA	1.50	0.90,2.60	NA; NA	NA
Breast Cancer	vitamin E supplements	Fulan et al. (26)	NA	12	NA	NA	NA	OR	Random	0.98	0.92,1.04	0.0%; NA	0.010
Breast Cancer	combined intake vitamin E	Fulan et al. (26)	NA	14	NA	NA	NA	OR	Random	0.82	0.73,0.91	44.2%; NA	1.000
Colorectal Cancer	dietary vitamin E	Liu (36)	NA	13	NA	NA	NA	RR	Random	0.94	0.82,1.07	10.3%; NA	0.018
Kidney Cancer	dietary vitamin E	Shen et al. (31)	5,731/ 20,543	7	0	7	0	RR	NA	0.78	0.62,0.97	49.2%; 0.023	0.928
Endometrial Cancer	dietary vitamin E	Bandera et al. (37)	2,800/ 3,873	6	0	6	0	OR	Random	0.91	0.84,0.99	0.0%; 0.450	NA
Ovarian Cancer	total intake vitamin E	Leng et al. (38)	4,597/ 440,532	14	5	9	0	RR	Random	0.95	0.78,1.16	53.2%; 0.019	NA
NHL	total intake vitamin E	Psaltopoulou et al. (39)	3,840/ 189,522	4	4	0	0	RR	Random	0.94	0.65,1.36	0.0%; 0.889	NA
Total Cancer	dietary vitamin E	Aune et al. (40)	5,718/ 169,236	5	NA	NA	NA	RR	Random	0.97	0.93,1.02	61.0 %; 0.030	0.070
Cancer Mortality	dietary vitamin E	Schwingshackl et al. (41)	3,605/ 279,666	3	NA	NA	NA	RR	Fixed	1.00	0.79,1.28	45.0%; NA	NA

CI, confidence interval; RCT, randomized controlled trial; RR, relative risk; OR, odds ratio; NA, not available; NHL, non-Hodgkin lymphoma.

**Table 3:** Assessments of AMSTAR scores and GRADE classification.

**Outcome**	**Category**	**Author**	**Year**	**AMSTAR**	**GRADE**
**Non-cancer outcomes**				
**Significant associations**				
Cardiovascular disease	Vitamin E supplements	Han	2020	10	Low
Parkinson's Disease	Dietary vitamin E	Talebi	2022	8	Very low
Depression	Dietary vitamin E	Lee	2022	8	Very low
Anxiety	Dietary vitamin E	Lee	2022	8	Very low
Age-related Cataract	Dietary vitamin E	Jiang	2019	9	Very low
Metabolic Syndrome	Dietary vitamin E	Zhang	2021	9	Very low
Fracture	Dietary vitamin E	Zhou	2020	9	Low
**Non-significant associations**				
Stroke	Vitamin E supplements	Loh	2021	9	Moderate
Parkinson's Disease	Dietary vitamin E	Talebi	2022	8	Very low
Alzheimer's Disease	Vitamin E supplements	Wang	2021	9	Very low
Obesity	Vitamin E supplements	Mohammad	2021	10	Moderate
Glaucoma	Dietary vitamin E	Han	2022	7	Very low
Age-related Cataract	Dietary vitamin E	Jiang	2019	9	Moderate
All-Cause Mortality	Dietary vitamin E	Jayedi	2018	7	Low
**Cancer outcomes**				
**Significant associations**				
Breast Cancer	Total intake vitamin E	Fulan	2011	10	Low
Breast Cancer	Dietary vitamin E	Fulan	2011	10	Low
Lung Cancer	Dietary vitamin E	Zhu	2017	7	Low
Esophageal Cancer	Dietary vitamin E	Cui	2018	7	Very low
Gastric Cancer	Dietary vitamin E	Kong	2014	8	Low
Pancreatic Cancer	Dietary vitamin E	Chen	2016	9	Low
Kidney Cancer	Dietary vitamin E	Shen	2015	8	Low
Bladder Cancer	Dietary vitamin E	Lin	2019	8	Low
Cervical Neoplasm	Total intake vitamin E	Hu	2017	8	Very low
**Non-significant associations**				
Glioma	Dietary vitamin E	Qin	2014	9	Very low
Thyroid Cancer	Dietary vitamin E	Zhang	2013	6	Very low
Breast Cancer	Combined intake vitamin E	Fulan	2011	10	Low
Breast Cancer	Combined intake vitamin E	Fulan	2011	10	Low
Colorectal Cancer	Dietary vitamin E	Liu	2015	9	Very low
Kidney Cancer	Dietary vitamin E	Shen	2015	8	Low
Endometrial Cancer	Dietary vitamin E	Elisa	2008	6	Low
Ovarian Cancer	Total intake vitamin E	Leng	2019	9	Very low
NHL	Total intake vitamin E	Psaltopoulou	2018	5	Very low
Total Cancer	Dietary vitamin E	Aune	2018	7	Low
Cancer Mortality	Dietary vitamin E	Schwingshackl	2017	8	Very low

AMSTAR, a measurement tool to assess systematic reviews; GRADE, Grading of Recommendations Assessment, Development, and Evaluation; NHL, non-Hodgkin lymphoma.

### 3.2. Associations between VE intake and cancer outcomes

Comparing “the highest” with “the lowest” intake, total intake of VE and dietary intake of VE significantly reduced the risk of breast cancer by 11% (OR = 0.89, 95% CI: 0.81,0.97) and 18% (OR = 0.82, 95% CI:0.73,0.91), respectively (26). The highest dietary VE intake was significantly associated with a decreased risk of lung cancer (RR = 0.84, 95% CI = 0.76,0.93). Subgroup analysis by geographic location showed significant negative associations between dietary VE intake and the risk of lung cancer for the American and European populations (RR = 0.85, 95% CI = 0.75,0.95) but not for the Asian population. Note that there exists a linear relationship between dietary VE intake and lung cancer risk: a daily dietary intake of 2 mg of VE reduces the risk by 5% (27).

Higher dietary VE was also related to lower esophageal cancer risk (OR = 0.47, 95% CI: 0.36, 0.60), especially for esophageal squamous cell carcinoma (ESCC) (OR = 0.29, 95% CI: 0.18, 0.44). A slightly linear dose–response relationship was detected between a 3 mg/day increment of dietary VE and the risk of esophageal cancer (OR = 0.78; 95% CI: 0.57, 1.06) (28). Furthermore, a dose–response relationship was detected between 10 mg/day of dietary VE intake and gastric cancer risk (RR=0.76, 95% CI: 0.67, 0.85) (29). A significant association was found between VE intake and pancreatic cancer risk for only case-control studies (pooled OR=0.63, 95% CI 0.53, 0.75). Meanwhile, a subgroup analysis based on the geographic area found that the intake of VE was not significantly associated with pancreatic cancer risk in European countries, while the inverse association was found in other geographic areas (30).

An analysis of the highest vs. lowest VE intake revealed that the intake of VE played a protective role in bladder cancer progression (RR=0.89; 95% CI: 0.78,1.00). Moreover, a potential linear association was also detected between VE intake and bladder cancer risk (32). A significant negative association between VE intake and kidney cancer risk was found only for cohort studies (RR=0.88, 95% CI 0.72,1.08) (31). In addition, VE intake also has a significant inverse association with the risk of cervical neoplasia (OR=0.68; 95% CI: 0.49, 0.94) (33).

No significant association was observed between VE consumption and the risks of glioma (34), thyroid cancer (35), colorectal cancer (36), endometrial cancer (37), ovarian cancer (38), and non-Hodgkin lymphoma (NHL) (39). Furthermore, the association between VE and the risk of overall cancer mortality (41) or total cancer (40) is not significant. Additionally, no significant association was detected between the combined intake of VE or VE supplements and breast cancer. Additionally, when we performed a subgroup analysis based on the type of study, the relationship between VE intake and breast cancer or pancreatic cancer was not significant (26, 30). For kidney cancer, the association was non-significant in case-control studies (31).

### 3.3. Associations between VE intake and non-cancer outcomes

A higher intake of VE supplements was associated with a significant reduction in the risk of cardiovascular disease (RR=0.92; 95% CI: 0.46, 0.95) (15). Compared with the lowest category of dietary VE intake, the highest dietary VE intake was significantly associated with a 16% lower risk of Parkinson's disease in the analysis of cohort studies (RR= 0.84; 95% CI: 0.71, 0.99). A linear dose–response meta-analysis suggested that each 5 mg/day increment in VE intake was associated with a 16% lower risk of Parkinson's disease (16). In adults who are at risk of or clinically diagnosed with depression, the positive effect of VE supplements on mood outcomes was observed (SMD = −0.88; 95% CI: −1.54, −0.21) (17). A 10% reduction in the risk of age-related cataracts for individuals was found in the highest categories of VE supplements for cohort studies (RR = 0.90; 95% CI: 0.80, 1.00) (18). In addition, dietary VE intake was inversely associated with a lower risk of metabolic syndrome (MetS) for high versus low consumption (SMD = −0.08; 95% CI: −0.14, −0.02) (19). In terms of obesity, subgroup analysis for baseline body mass index (BMI) suggested that VE supplements had a significant effect on increasing BMI in participants with normal baseline BMI (18.5–24.9) (WMD=0.636; 95% CI: 0.01, 1.26) (24). Furthermore, the risk of fracture at all sites was significantly reduced with higher VE intake (RR = 0.66; 95% CI: 0.46,0.95), especially for men (20).

Dietary VE intake was not associated with the risk of all-cause mortality while comparing the highest group with the lowest group (RR= 0.95; 95% CI: 0.90, 1.01), even in the further subgroup analysis (25). Higher intake of VE supplements did not show a significant association with stroke (21), anxiety (17), or Alzheimer's disease (22). Dietary VE intake was also associated with a lower risk of glaucoma (23), but it was not statistically significant. In addition, the subgroup analysis did not present a significant association between dietary VE intake and the risk of Parkinson's disease in case-control studies (16), and VE supplements had no significant effect on age-related cataract risk in RCTs (18).

### 3.4. Heterogeneity and publication bias of included meta-analyses

Among the 28 non-overlapping meta-analyses, 13 meta-analyses reported a Q test *P* < 0.10. One meta-analysis did not report the I^2^ statistic. A very high level of heterogeneity (I^2^>70%) was observed in three meta-analyses, and eight meta-analyses reported moderate-to-high levels of heterogeneity (I^2^ 50%−70%). Sixteen meta-analyses reported low levels of heterogeneity (I^2^ < 50%).

### 3.5. AMSTAR and GRADE evaluation of included meta-analyses

The quality of the evidence by GRADE was low (47.1%) or very low (44.1%) (Table 3). Three outcomes (stroke, age-related cataracts, and obesity) were identified as having a “moderate” level of quality. The AMSTAR scores of all health outcomes ranged from 5 to 10, with a median score of 8 (IQR 7-9) (Table 3). More details are presented in Supplementary Tables S1, S2.

## 4. Discussion

The associations between VE and multiple health outcomes have been reported by a large number of studies and integrated into many meta-analyses. Overall, 27 meta-analyses involving 28 unique outcomes of the correlation between VE intake and multiple health outcomes were included in this umbrella review. The results indicated that the intake of VE was related to a lower risk of subsequent cancer outcomes (breast cancer, lung cancer, esophageal cancer, gastric cancer, pancreatic cancer, kidney cancer, bladder cancer, and cervical neoplasms) and non-cancer outcomes (cardiovascular disease, Parkinson's disease, depression, age-related cataract, metabolic syndrome, and fracture). Given that most of the evidence was from observational studies, compelling evidence for VE and multiple health outcomes does not seem to exist.

There is some discrepancy in subgroup analyses based on the study type of several outcomes (breast cancer, pancreatic cancer, kidney cancer, Parkinson's disease, and age-related cataract). Some of the potential reasons are mentioned below. First, the development of some diseases, such as age-related cataracts, is a long process for healthy individuals, and it may take a decade to manifest the protective effect of VE intake (42). The lengths of follow-up in RCTs may not be long enough to observe the effects of VE supplementation on the risks of disease (43). Second, the dose of VE intake in RCTs, cohort studies, and case-control studies was different (44). Third, due to the different levels of difficulty associated with conducting different studies, there are generally more case-control studies than cohort studies and RCTs. However, for the retrospective design of the case–control studies, there was more selection and recall bias for VE intake measurement in the case-control studies (30). There were also discrepant findings in subgroup analyses of the type of VE intake. Most VE supplements were synthetic and only contained the α-tocopherol form of the eight isoforms found in natural VE. Thus, the natural form, taken in dietary form, shows a more pronounced protective effect than the synthetic form (45). This may explain the non-significant association between VE supplements or the combined intake of VE and breast cancer (26). Another possible reason may be that VE supplements are generally used by those who are more concerned about their health condition than others, which means that they are more likely to tend to adopt a healthier lifestyle (45), which may also lead to discrepancies in the results.

The results of this study suggested that higher VE intake is negatively associated with the risk of cancer outcomes (breast cancer, gastric cancer, pancreatic cancer) and non-cancer outcomes (cardiovascular disease, Parkinson's disease, depression, age-related cataract). The protective effect of higher VE intake on these outcomes may be explained by the oxidative properties of VE. A possible mechanism might be that, as an antioxidant, VE can prevent DNA from being damaged by scavenging lipid hydrogen peroxide radicals (44, 46, 47). Moreover, VE could activate apoptosis by repressing the protein kinase C (PKC) pathway, enhancing immune system function, and inhibiting cancer cell growth by decreasing the phosphoinositide 3-kinase pathway (48). Recent research found that dietary VE could inhibit the dendritic cell checkpoint SHP1 from boosting antigen presentation, strengthening antitumor T-cell immunity, and enhancing immunotherapy (49). In addition, inhibiting cell cycle progression and cell proliferation via the reduction of cyclin D1 and cyclin E could be a feasible explanation for the protective effect of VE against lung cancer and bladder cancer (50, 51). The functions of inhibiting peroxidation and inducing cell apoptosis and, in turn, leading to the suppression of cell proliferation by effectively decreasing the expression of cyclooxygenase-2 and 8-hydroxydeoxyguanosine and type I insulin-like growth factor receptors may play an important role in the significant inverse association of VE intake and kidney cancer risk (52). In previous studies, VE also displayed neuroprotective functions against free radical-mediated injury. This is exemplified by its protection of neurons in the locus coeruleus (the main site of norepinephrine synthesis) from death in an early model of Parkinson's disease, preventing the toxin-induced damage of striatal dopaminergic terminals, and controlling the functions of antioxidant defenses, such as glutathione and superoxide dismutase (SOD) (53–55). Furthermore, to maintain the integrity of proteins and membranes and mediate the function of the lens, VE plays a vital role in blocking the excessive activation of oxidative stress (56–58). These findings can also explain the reverse association, which is a dynamic and interactive process, between dietary VE intake and metabolic syndrome (MetS) (59, 60). Although the exact mechanism of VE supplements' effects on body mass indices (BMI) has not yet been detected, the role of VE on the activation of peroxisome proliferator-activated receptor gamma (PPARg), which could lead to the upregulation of adiponectin gene expression, could be a possible pathway. Moreover, improving insulin sensitivity and suppressing HMG-CoA reductase could also be possible mechanisms affecting body composition indicators (61–63).

For the safety of VE supplements, healthy individuals should not use more than 1,000 mg of VE per day. A daily intake of VE of up to 800 IU appears safe and beneficial (64). When the intake of VE reaches between 400 and 800 IU, healthy individuals appear to have a decreased risk of several diseases, such as CVDs (65). However, the intake of VE could promote the degradation of essential medications for conditions such as cancer, cardiovascular diseases, hypertension, or diabetes. Although a study conducted by Podszun and Frank did not find evidence for the interaction between tocopherols or tocotrienols in the body, it has been suggested that exceeding dosages of 300 mg/day may interfere with some xenobiotics, such as tamoxifen, cyclosporine A, aspirin, or warfarin (66). Under these special circumstances, it is necessary to seek the opinion of a specialist regarding the need and appropriate dosage of vitamin E supplementation. In short, to date, many studies have found potential benefits of VE for multiple disease risks, but no study suggests a specific dose or appropriate population. For healthy individuals, 1,000 mg a day is the upper limit, but more research is needed to determine whether regular supplements are recommended and how much they should be given to specific populations, such as those at high risk for disease and cancer. Prophylactic use of large doses of vitamin E supplements (>1,000 mg per day) is not recommended. When some drugs are being used, the dose of VE should be more carefully designed.

Notably, this is the first comprehensive review of available evidence on VE intake and multiple health outcomes. Standard tools were used to assess the quality of the methods in the included literature (AMSTAR) and the strength of the evidence (GRADE). To avoid possible selection bias, the study was conducted by two researchers. However, our study has several limitations. First, most of the meta-analyses included were based on retrospective studies, and the overall GRADE quality was low. Second, considering that the most common source of VE is dietary intake, it is difficult to obtain VE as the only antioxidant or key nutrient. Third, the results of the study have a large deviation, and there are more confounding factors. Micronutrient combinations have also been frequently used in studies where the interaction between multivitamins is not further elucidated, and not all meta-analyses have a subgroup analysis of these factors. Finally, the definitions of maximum and minimum intakes were not clearly and uniformly quantified, making it difficult to determine the magnitude of the correlation and the impact of standardized baselines, and dose–response analyses were performed in less than half of the included meta-analyses.

## 5. Conclusion

In conclusion, we concluded that the intake of VE was related to a lower risk of multiple types of cancer and other diseases of diverse systems. Thus, VE intake at a safe dose is recommended to gain protect against certain diseases. However, further high-quality studies on recommended doses and recommended populations of VE are also needed. For specific populations (such as patients with high blood pressure, diabetes, and cancer) who are taking medication, additional vitamin E supplementation needs to be evaluated by a specialist before use.

## Data availability statement

The original contributions presented in the study are included in the article/Supplementary material, further inquiries can be directed to the corresponding author.

## Author contributions

JA and TZ conceived the project and drafted the manuscript. TZ, XY, JL, and XZ performed the screening and extraction. DL and HX performed the statistical analysis. JA revised the manuscript. All authors contributed to the article and approved the submitted version.
